# Neuromechanics-Based Neural Feedback Controller for Planar Arm Reaching Movements

**DOI:** 10.3390/bioengineering10040436

**Published:** 2023-03-30

**Authors:** Yongkun Zhao, Mingquan Zhang, Haijun Wu, Xiangkun He, Masahiro Todoh

**Affiliations:** 1 Division of Human Mechanical Systems and Design, Graduate School of Engineering, Hokkaido University, Sapporo 060-8628, Japan; 2 Division of Bioengineering, Graduate School of Engineering Science, Osaka University, Osaka 560-8531, Japan; 3 State Key Laboratory of Bioelectronics, Jiangsu Provincial Key Laboratory of Remote Measurement and Control, School of Instrument Science and Engineering, Southeast University, Nanjing 210096, China; 4 Division of Mechanical and Aerospace Engineering, Faculty of Engineering, Hokkaido University, Sapporo 060-8628, Japan; 5 Department of Bioengineering, Faculty of Engineering, Imperial College London, London SW7 2AZ, UK

**Keywords:** neuromechanics, musculoskeletal arm, neural feedback controller, arm reaching movement

## Abstract

Based on the principles of neuromechanics, human arm movements result from the dynamic interaction between the nervous, muscular, and skeletal systems. To develop an effective neural feedback controller for neuro-rehabilitation training, it is important to consider both the effects of muscles and skeletons. In this study, we designed a neuromechanics-based neural feedback controller for arm reaching movements. To achieve this, we first constructed a musculoskeletal arm model based on the actual biomechanical structure of the human arm. Subsequently, a hybrid neural feedback controller was developed that mimics the multifunctional areas of the human arm. The performance of this controller was then validated through numerical simulation experiments. The simulation results demonstrated a bell-shaped movement trajectory, consistent with the natural motion of human arm movements. Furthermore, the experiment testing the tracking ability of the controller revealed real-time errors within one millimeter, with the tensile force generated by the controller’s muscles being stable and maintained at a low value, thereby avoiding the issue of muscle strain that can occur due to excessive excitation during the neurorehabilitation process.

## 1. Introduction

The social aging of the population is currently one of the greatest concerns facing nations worldwide [1]. The number of elderly people with spinal cord injuries, Parkinson’s disease, and other diseases is constantly rising [2,3,4]. The most noticeable symptom of these age-related diseases is tremor, which prevents older people from performing motor activities and stabilizing desired movements [5]. There are many techniques that have been developed to address this kind of issue, and the most welcomed therapy adopted by medical professionals is functional electrical stimulation (FES) [6]. FES is an effective therapy for patients who have lost the ability to perform desired motions, which generates body movements artificially by using low-energy electrical pulses [7]. This therapy was first proposed by Liberson and his co-workers in the year 1961 and aimed to stimulate the peroneal nerve to correct foot drop by triggering a foot switch [8]. When using FES on patients, different electrical currents of varying number and intensity produce diverse results [9]. Hence, how to intelligently activate the FES device to provide the electrical pulses has attracted the attention of numerous researchers.

To achieve high-precision, task-specific FES rehabilitation therapy, many researchers started to investigate the mechanism of how the central nervous system (CNS) can control the human body to complete a specific motor task and tried to utilize the FES technique to generate the motion based on this mechanism. For the human arm reaching movement, Bernstein first described this movement in terms of coordinates, which served as the foundation for a qualitative and quantitative analysis of its kinematics and dynamics [10]. Apart from the investigation from a mathematical aspect, Morraso et al. conducted numerous experiments and the results suggest that human arm movement trajectories are planned in task-oriented coordinates rather than joint coordinates [11]. Specifically, human arm reaching movement trajectories from the start to the desired position are in the shape of roughly a straight path, and the speed profile of that is in the shape of a bell curve. Subsequently, investigations on the analysis of biomechanical structure, modeling, and development of motor control, etc. for the human arm were sparked. By building a model of the human arm, Uno et al. for the first time analyzed its kinematics and dynamics, providing the theoretical underpinnings for the motor control of arm reaching actions [12]. Based on these findings, Jagodnik et al. concluded that the feedback gains function in the brain to control joint angles and produce the required movements [13]. However, because the controller was based on a skeleton arm, none of them gave adequate consideration to the influence of muscles during designing the motor controller. Thomas et al. used an actor–critic architecture in the motor controller to cause the skeletal muscles to contract, causing the arm to move [14]. To control the movement of a musculoskeletal arm, Tahara et al. used an iterative learning approach [15]. A fuzzy sliding controller for this musculoskeletal arm was created by Chen et al. to conduct reaching movements [16]. Hausmann et al. used neural networks to simulate the sensorimotor system of the brain and create smooth trajectories that resembled the actual arm reaching action [17]. However, all of their designs of motor controllers stray from the fundamentals of neuroscience and are solely intended to drive the arm from one position to another. According to neuroscience studies, the many functional regions of the brain control the arm’s natural movement trajectory [18]. Therefore, it is crucial to develop a motor controller for FES that is neurophysiology-based, human brain-like, and has multifunctional sections. Apart from the application for FES, this kind of controller can be applied to many other fields such as the energy sector [19], flight control [20], vehicle technologies [21], etc.

To address this issue, in this paper, a neuromechanics-based neural feedback controller for arm reaching movements is developed. Compared to the research mentioned above, the contribution of this paper is threefold. First, the controller is developed based on neuromechanics which deems the motion is generated through the interaction among neural, muscular, and skeletal systems. In detail, this controller transits the neural signals to skeletal muscles, and then the skeletal muscles generate the tensile forces. After that, the skeletons are driven by these tensile forces from the start position towards the target position. This sort of controller operating mechanism is the first one to be employed for arm reaching movements. Second, as the controller is designed based on neuromechanics, a model that mimics the real human arm is needed. Here, a musculoskeletal arm model is constructed, consisting of two skeletons and seven muscles, each of which is modeled after the genuine skeletons and muscles. Third, to prevent the patients from serious muscle strain during arm reaching movements, the controller stimulates the muscles to generate small and steady forces, which are sufficient to drive the arm toward the target position. In other words, with this controller, not too much energy consumption is required to perform arm reaching movements.

The structure of this paper is as follows: In Section 2, the kinematical and dynamical properties of the human musculoskeletal arm are modeled. Section 3 describes the mechanism of the neuromechanics-based neural feedback controller. Section 4 presents the numerical simulation experiments to validate the good performance of the controller. Section 5 concludes this paper with a summary of the results and discusses the foreseen challenges for the application of neural feedback controllers.

## 2. Musculoskeletal Arm Model

**Figure 1:**
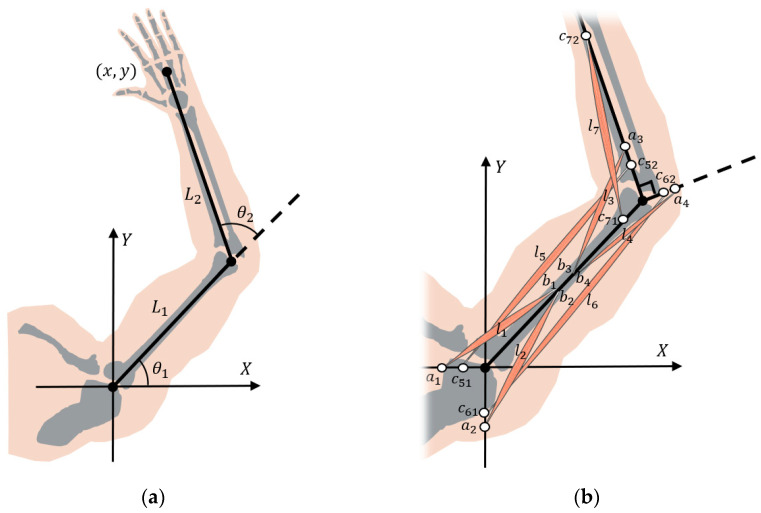
Schematic diagram of the human musculoskeletal arm model: (**a**) a planar kinematic model of the human arm; (**b**) a planar dynamic model of the human musculoskeletal arm.

To design a good-performance controller for arm reaching movements, a precise computational model should be constructed. Following the definition of neuromechanics, motions are generated through the interactions among the neural, muscular, and skeletal systems. As people with SCI cannot transmit their neural signals to the muscles, the effect of the neural system is not necessary to consider. Therefore, only the effects of muscles and skeletons are assimilated into this model. In detail, this model comprises two skeletal links and seven skeletal muscles. Two skeletal links represent the real upper limb and forearm, while seven skeletal muscles represent the seven main muscles contributing to arm reaching movements, which are the biceps brachii (BB), triceps brachii (TB), brachialis (BRA), and brachioradialis (BRD). It should be noted that the BB is composed of two muscles, which are the biceps long head (BIClong) and biceps short head (BICshort). The TB is composed of three muscles, which are the triceps lateral head (TRIlat), triceps long head (TRIlong), and triceps medial head (TRImed). This musculoskeletal arm model is validated in our previous paper [16,22]. The anatomy drawings and schematic diagrams of the arm are illustrated in Figure 1.

### 2.1. Kinematics of Skeletons

First, the kinematics of the skeletons is analyzed. The end position of the arm, represented as a vector, can be obtained via the forward kinematics analysis [23]:

(1) $$\begin{array}{c} P=\left[\begin{array}{c} x\\ y \end{array}\right]=\left[\begin{array}{c} L_{1}\cos\theta_{1}+L_{2}\cos\left(\theta_{1}+\theta_{2}\right)\\ L_{1}\sin\theta_{1}+L_{2}\sin\left(\theta_{1}+\theta_{2}\right) \end{array}\right]\; \end{array}$$

where P represents the end position of the arm, $L_{1}$ and $L_{2}$ are the lengths of the upper limb and forearm, respectively, $\theta_{1}$ is the angle between the upper limb and x axis, while $\theta_{2}$ is the angle between the forearm and the central axis of the upper limb. 

Through conducting the inverse kinematics on Equation (1), the angles $\theta_{1}$ and $\theta_{2}$ can be derived as follows [24]:

(2) $$\theta =\left[\begin{array}{c} \theta_{1}\\ \theta_{2} \end{array}\right]=\left[\begin{array}{c} \arctan\frac{y}{x}-\arctan\frac{L_{2}\sin\theta_{2}}{L_{1}+L_{2}\cos\theta_{2}}\\ \arccos\frac{x^{2}+y^{2}-L_{1}^{2}-L_{2}^{2}}{2L_{1}L_{2}} \end{array}\right]$$

The velocity of the end position of the arm can be obtained by calculating derivatives of Equations (1) and (2) concerning time [25]:

(3) $$\dot{P}=J\dot{\theta }\;$$

(4) $$\dot{P}=\left[\begin{array}{c} \dot{x}\\ \dot{y} \end{array}\right]\;$$

(5) $$J=\left[\begin{array}{cc} -L_{1}\sin\theta_{1}-L_{2}\sin\left(\theta_{1}+\theta_{2}\right) & -L_{2}\sin\left(\theta_{1}+\theta_{2}\right)\\ L_{1}\cos\theta_{1}+L_{2}\cos\left(\theta_{1}+\theta_{2}\right) & L_{2}\cos\left(\theta_{1}+\theta_{2}\right) \end{array}\right]\;$$

(6) $$\dot{\theta }=\left[\begin{array}{c} {\dot{\theta }}_{1}\\ {\dot{\theta }}_{2} \end{array}\right]\;\;$$

where $\dot{P}\in {\mathbb{R}}^{2\times 1}$ is the velocity of the end position of the arm with respect to the x axis and y axis, $\dot{\theta }\in {\mathbb{R}}^{2\times 1}$ is the angular velocity of the shoulder joint and elbow joint, and $J\in {\mathbb{R}}^{2\times 2}$ is the Jacobian matrix which represents the relationship between the linear velocity of the end position of the arm and the angular velocity of joints.

### 2.2. Kinematics of Skeletal Muscles

Two assumptions are given here. First, the skeletal muscles are assumed to contract linearly due to no flexible deformation. Second, the effect of the mass transfer of the skeletal muscles is not considered as it does not take an important role during arm reaching movements. Based on these two assumptions, the lengths of each muscle can be defined as follows [26]:

(7) $$l={\left[l_{1},\;l_{2},l_{3},l_{4},l_{5},l_{6},l_{7}\right]}^{T}\#$$

(8) $$\left[\begin{array}{c} l_{1}\\ l_{2}\\ \begin{array}{c} l_{3}\\ l_{4}\\ \begin{array}{c} l_{5}\\ \begin{array}{c} l_{6}\\ l_{7} \end{array} \end{array} \end{array} \end{array}\right]=\left[\begin{array}{c} {\left(a_{1}{}^{2}+b_{1}{}^{2}+2a_{1}b_{1}\cos\theta_{1}\right)}^{\frac{1}{2}}\\ {\left(a_{2}{}^{2}+b_{2}{}^{2}-2a_{2}b_{2}\cos\theta_{1}\right)}^{\frac{1}{2}}\\ \begin{array}{c} {\left(a_{3}{}^{2}+b_{3}{}^{2}-2a_{3}b_{3}\cos\theta_{2}\right)}^{\frac{1}{2}}\\ {\left(a_{4}{}^{2}+b_{4}{}^{2}-2a_{4}b_{4}\cos\theta_{2}\right)}^{\frac{1}{2}}\\ \begin{array}{c} {\left(c_{51}{}^{2}+c_{52}{}^{2}+L_{1}{}^{2}+2c_{51}L_{1}\cos\theta_{1}+2c_{52}L_{1}\cos\theta_{2}+2c_{51}c_{52}\cos\left(\theta_{1}+\theta_{2}\right)\right)}^{\frac{1}{2}}\\ \begin{array}{c} {\left(c_{61}{}^{2}+c_{62}{}^{2}+L_{1}{}^{2}-2c_{61}L_{1}\cos\theta_{1}-2c_{62}L_{1}\cos\theta_{2}+2c_{61}c_{62}\cos\left(\theta_{1}+\theta_{2}\right)\right)}^{\frac{1}{2}}\\ {\left(c_{71}{}^{2}+c_{72}{}^{2}+L_{2}{}^{2}+2c_{71}L_{2}\cos\theta_{2}+2c_{72}L_{2}+2c_{71}c_{72}\cos\left(\theta_{1}+\theta_{2}\right)\right)}^{\frac{1}{2}} \end{array} \end{array} \end{array} \end{array}\right]$$

where $a_{1-4}$, $b_{1-4}$, $a_{51}$, $a_{52}$, $a_{61}$, $a_{62}$, $b_{61}$, and $b_{62}$ are the moment levers of each skeletal muscle, which follows the assumption that the values of them are constant during the arm reaching movements. In detail, the matrix of moment levers is defined as follows [27]:

(9) $$q={\left[\begin{array}{c} -a_{1}\\ 0 \end{array}\;\;\;\begin{array}{c} a_{2}\\ 0 \end{array}\;\;\;\begin{array}{c} 0\\ a_{3} \end{array}\;\;\;\begin{array}{c} 0\\ a_{4} \end{array}\;\;\;\begin{array}{c} -c_{51}\\ -c_{52} \end{array}\;\;\;\begin{array}{c} c_{61}\\ c_{62} \end{array}\;\;\;\begin{array}{c} -c_{71}\\ -c_{72} \end{array}\right]}^{T}$$

Through deriving Equation (7) concerning time, the following equation can be obtained [28]:

(10) $$\dot{l}=W\dot{\theta }\;$$

where $W\in {\mathbb{R}}^{6\times 2}$ is the Jacobian matrix which represents the relationship between the velocity of muscle contraction and the angular velocity of joints. 

### 2.3. Dynamics of Skeletons

The dynamics of the skeletons can be derived from the Lagrangian equation of motion [29]:

(11) $$M\left(\theta \right)\ddot{\theta }+C\left(\theta ,\dot{\theta }\right)\dot{\theta }+V\dot{\theta }=\tau \;$$

where M, C, and V are inertia, Coriolis, and viscosity matrix, respectively. The details of them are defined as follows [30]:

(12) $$M\left(\theta \right)=\left[\begin{array}{cc} \alpha +2\beta \cos\theta_{2} & \delta +\beta \cos\theta_{2}\\ \delta +\beta \cos\theta_{2} & \delta \end{array}\right]\;$$

(13) $$C\left(\theta ,\dot{\theta }\right)=\left[\begin{array}{cc} -\beta {\dot{\theta }}_{2}\sin\theta_{2} & -\beta \left({\dot{\theta }}_{1}+{\dot{\theta }}_{2}\right)\sin\theta_{2}\\ \beta {\dot{\theta }}_{1}\sin\theta_{2} & 0 \end{array}\right]$$

(14) $$V=\left[\begin{array}{cc} v_{11} & v_{12}\\ v_{21} & v_{22} \end{array}\right]\;$$

where the details of α, β, and δ are defined as follows:

(15) $$\alpha =I_{1}+I_{2}+m_{1}L_{g1}^{2}+m_{2}\left(L_{1}^{2}+L_{g2}^{2}\right)\;$$

(16) $$\beta =m_{2}L_{1}L_{g2}\;$$

(17) $$\delta =I_{2}+m_{2}L_{g2}^{2}\;$$

where $m_{1}$ and $m_{2}$ are the mass of the upper arm and forearm, $I_{1}$ and $I_{2}$ are the inertia of them, and $L_{1}$, $L_{1}$ and $L_{g1}$, $L_{g2}$ are the length and the center length of the mass of them.

### 2.4. Dynamics of Skeletal Muscles

**Figure 2:**
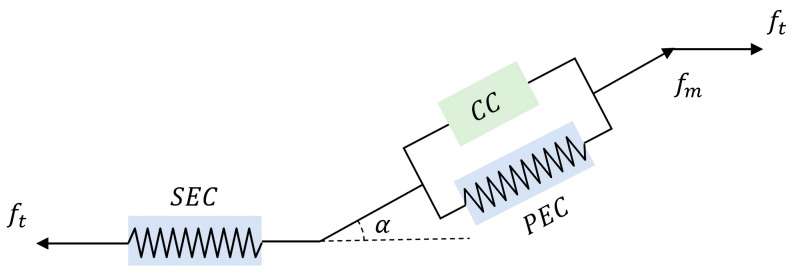
The diagram of the Hill-based muscle model, which is composed of the CC, PEC, and SEC [19], where $f_{t}$ and $f_{m}$ are the muscle tendon force and muscle fiber force, and α is the pennation angle, which is the angle between the direction of muscle fibers and the direction of muscle behaviors.

The behavior of skeletal muscles is strongly nonlinear; to model it, a Hill-based muscle modeling method is used here, which is validated for a good description of the force–velocity relation of skeletal muscle by our previous study [19]. The Hill-based muscle model consists of three parts: contractile, series elastic, and parallel elastic components. The contractile component (CC) is in series with the series elastic component (SEC), and both are parallel to the parallel elastic component (PEC). The arrangement of them is visualized in Figure 2.

#### 2.4.1. Contractile Component

A motor neuron is stimulated, which causes it to produce and transmit a neurological signal to the muscular system. The total of the motor unit action potentials is the signal, which is categorized by the Hill model as a time-varying signal. Additionally, the value of the signal is set to range from 0 to 1. Following receipt of this signal, the CC turns on and transmits the signal to the activation level, which also has a value range of 0 to 1. The force generated by the CC component is determined by three factors, which are the activation level, force–length relationship, and force–velocity relationship. The equation for it is defined as follows [31]:

(18) $$f_{cc}=f_{0}\;\cdot a\cdot f_{fl}\cdot f_{fv}$$

where $f_{cc}$ represents the CC force, $f_{0}$, $f_{fl}$, and $f_{fv}$ are the maximum isometric force, the relationship of force–length, and force–velocity, respectively, and α denotes the activation level.

#### 2.4.2. Series Elastic Component

The SEC describes the force–extension relationship of the skeletal muscle. The force generated by the SEC is commonly thought to be zero when its length is shorter than its unloaded length. The SEC force gradually increases when its length is extended over the unloaded length during muscle contraction. The equation for it is expressed as follows [32]:

(19) $$f_{sec}=\{\begin{array}{cc} \gamma \left(l_{sec}-l_{u}\right) & \mathrm{if}\;l_{sec}>l_{u}\\ 0 & \mathrm{if}\;l_{sec}\leq l_{u} \end{array}$$

(20) $$\gamma =\frac{f_{0}}{{\left(u_{0}\cdot l_{u}\right)}^{2}}\;$$

where $l_{sec}$ and $l_{u}$ are the SEC length and the unloaded length, and $u_{0}$ is a constant value that determines the muscle energetics.

#### 2.4.3. Parallel Elastic Component

In addition to the CC and SEC, the PEC is another part of the Hill-based muscle model, and it is arranged parallel to the CC. The force produced by the PEC is dependent on the forces generated by the CC and SEC. The formula for it is described as follows [33]:

(21) $$f_{pec}cos\alpha =f_{sec}-f_{cc}cos\alpha$$

(22) $$f_{t}=f_{pec}+f_{sec}=f_{pec}+f_{cc}cos\alpha \;$$

where $f_{pec}$, $f_{sec}$, and $f_{t}$ are the PEC force, SEC force, and the total force exerting on the muscle tendon, respectively. 

## 3. Neural Feedback Controller

The neural feedback controller of the FES for assisting arm reaching movements is composed of three main parts, which are the fuzzy system, proportional and derivative (PD) feedback gains, and neural networks. The PD feedback gains have been demonstrated to be the natural feedback control policy adopted by the human brain [20,21]. While the fuzzy system is to imitate that the brain cannot accurately calculate the distance between the start and target position, instead, it calculates the distance in a fuzzy manner [22]. For the neural networks, it aims to decrease the energy consumption during performing the arm reaching movements by making the skeletal muscles generate relatively small tensile forces. With this neural feedback controller for FES, the arm can be driven from the start position to the target position smoothly. In this section, each part of this controller is described in detail. 

### 3.1. Fuzzy System

The fuzzy system in this controller is to imitate that the brain controls the arm to reach the target position via approximate sensory feedback on the distance between the current and target position. Such a mechanism is similar to that of the fuzzy system, which is composed of a fuzzification unit, fuzzy rule base, fuzzy inference engine, and defuzzification unit. The fuzzy system can be deemed as a nonlinear system that can map an input vector from $R^{n}$ to an output vector also from $R^{n}$. In detail, the fuzzifier maps the input vectors to fuzzy sets, and then the defuzzifier works in an opposite direction, i.e., it maps the fuzzy sets to output vectors. For the inference engine, its fuzzy rule base is the core component of the fuzzy system, which decides the accuracy and efficiency of computations. In this study, the input signals for the fuzzy system are the error signals and the changes of these error signals concerning a constant time span, while the output signals are the values of the PD feedback gains. The mathematical description of the input signals is given as follows [34]:

(23) $$e_{n}=\left[\begin{array}{c} e_{1_{n}}\\ e_{2_{n}} \end{array}\right]=\left[\begin{array}{c} \theta_{1_{n}}^{\prime }-\theta_{1_{n}}\\ \theta_{2_{n}}^{\prime }-\theta_{2_{n}} \end{array}\right]\;$$

(24) $$\Delta e_{n}=\left[\begin{array}{c} \Delta e_{1_{n}}\\ \Delta e_{2}{}_{n} \end{array}\right]=\left[\begin{array}{c} e_{1_{n}}-e_{1}{}_{n-1}\\ e_{2_{n}}-e_{2}{}_{n-1} \end{array}\right]\;$$

where $e_{n}$ is the nth joint angle error, including both the nth shoulder angle error $e_{1_{n}}$ and the nth elbow angle error $e_{2_{n}}$, $\theta_{1_{n}}^{\prime }$ and $\theta_{2_{n}}^{\prime }$ are the nth target shoulder and the nth elbow joint angle, $\theta_{1_{n}}$ and $\theta_{2}{}_{n}$ are the nth real shoulder and elbow joint, while $\Delta e_{n}$ is the nth change of joint angle error, including both the nth change of shoulder angle error $\Delta e_{1_{n}}$ and the nth change of elbow angle error $\Delta e_{2_{n}}$. $e_{1_{n-1}}$, $e_{1_{n}}$, $e_{2_{n-1}}$, and $e_{2_{n}}$ are the n-1th and nth change of shoulder angle error and elbow angle error.

**Figure 3:**
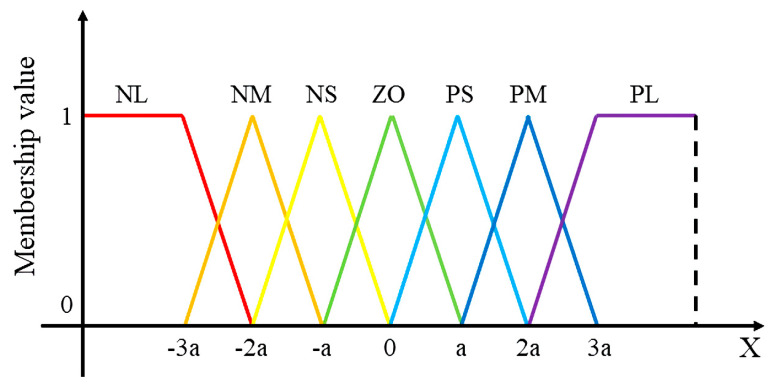
Membership functions of the fuzzy system, where a is set as 0.5 for joint angle error, while for the change of joint angle error, it is set as 0.01.

$e_{1_{n}}$, $e_{2_{n}}$, $\Delta e_{1_{n}}$, and $\Delta e_{2_{n}}$, as the input signals, are fed into the fuzzification interface, and then their own fuzzifications are obtained based on the membership function shown in Figure 3. After that, the fuzzifications of them are paired and given to the fuzzy inference engine. Then, the fuzzifications for the pairs are inferred based on the fuzzy rule base tables shown in Table 1 and Table 2. Finally, the inferred fuzzifications are transmitted into the defuzzification interface, and the update values of PD feedback gains are determined via the centroid defuzzification method.

### 3.2. Fuzzy PD System

Once the update values of the PD feedback gains are determined, the PD feedback gains for the controller can be obtained from the following equations [35]:

(25) $$K_{p_{n+1}}=K_{p_{n}}+\Delta K_{p_{n}}$$

(26) $$K_{d_{n+1}}=K_{d_{n}}+\Delta K_{d_{n}}\;$$

**Figure 4:**
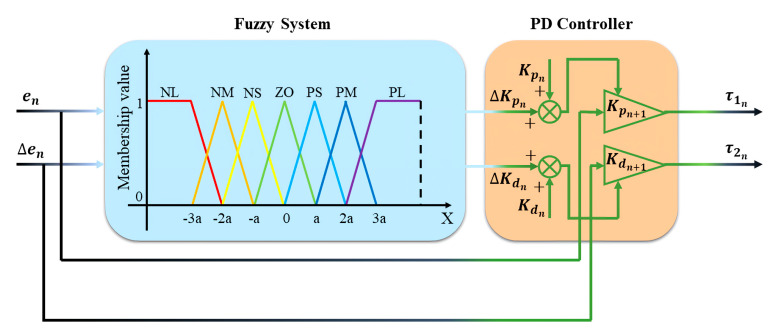
Fuzzy PD system of the neural feedback controller, which aims to generate the torques for driving the arm to the target position.

After that, the feedback gains are applied onto the equation of motion of the musculoskeletal arm to decrease the error between the current state and target state. Then, the torques needed for the upper limb and forearm are generated to drive the arm to the target position. The flow diagram of this process is shown in Figure 4. 

### 3.3. Neural Networks

**Figure 5:**
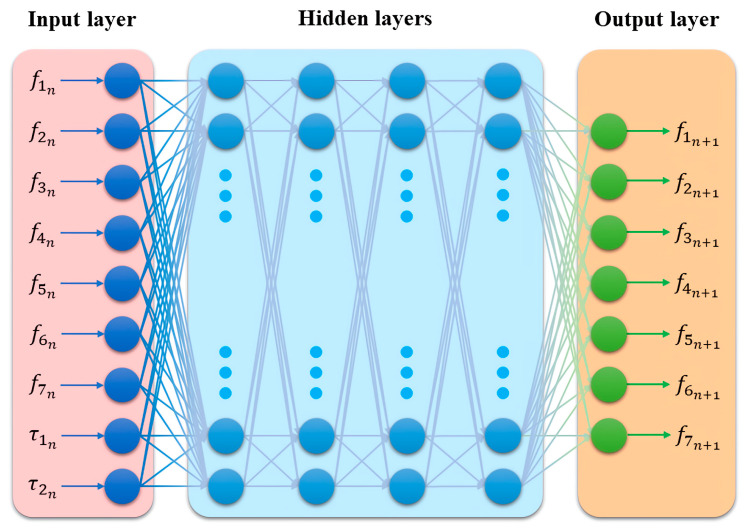
Neural networks of the neural feedback controller, which aim to generate the tensile forces for each skeletal muscle based on the torques needed for arm reaching movements.

The neural networks of this controller are to imitate the real activities of skeletal muscles during arm reaching movements. In detail, the skeletal muscles are activated in an antagonistic manner, and the tensile forces generated are limited to avoid muscle strain. These neural networks consist of three layers—input layer, hidden layer, and output layer—and each of them owns many processing elements. For the input layer, the inputs are given, while for the output layer, the outputs are generated. The hidden layer is responsible for processing the data from the input layer and transmitting the processing results to the output layer. In this study, the input vector is composed of nine elements, which are the tensile forces of the seven skeletal muscles, $f_{1}$, $f_{2}$, …, $f_{7}$, and the torques for the upper limb and forearm, $\tau_{1}$ and $\tau_{2}$. Two hidden layers, here, are used to extract the deep features of the data from the input layer. There is a total of 128 neurons in the hidden layers, which are sufficient to fit the real behaviors of skeletal muscles during arm reaching movements. After the data is processed by the hidden layers, the output layer generates the suitable tensile forces for each muscle. The neural networks of the neural feedback controller are shown in Figure 5. With these neural networks, the controller can determine the suitable tensile forces for each skeletal muscle based on the torques required to drive the arm to perform reaching movements. The skeletal muscles controlled by this controller behave in a manner that is comparable to skeletal muscles really activated by the brain. 

### 3.4. Adaptive Neuro-Fuzzy PD Controller

**Figure 6:**
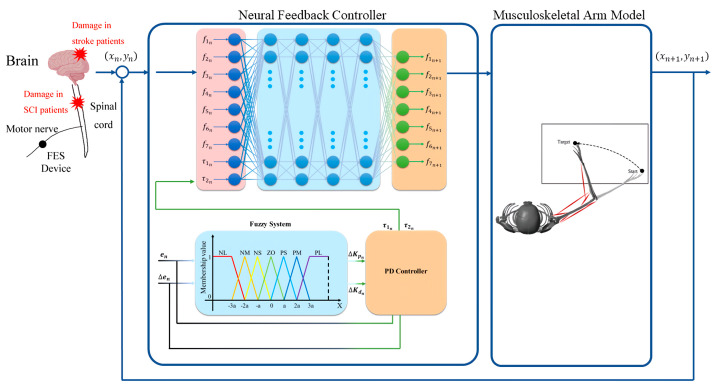
Block diagram of the neuromechanics−based neural feedback controller for arm reaching movements.

In this section, the components of the neural feedback controller described above are combined together. The block diagram of that is illustrated in Figure 6. As can be seen from the figure, the model, musculoskeletal arm consists of two skeletal links and seven skeletal muscles, which can be deemed as a hybrid nonlinear system. To control this system, at first, the fuzzy system outputs the update values of PD feedback gains $\Delta K_{p}$ and $\Delta K_{d}$ based on the input signals including the joint angle error e and the change of joint angle error Δe. Then, the PD gains $K_{p}$ and $K_{d}$ are updated, and the torques for the upper limb and forearm are determined. After that, the muscle tensile forces $f_{1},\;f_{2},\;\ldots \;,\;f_{7}$ and joint torques $\tau_{1}$ and $\tau_{2}$ are fed to the neural network, and it decides the new tensile forces for each muscle following the principle of antagonism. It should be noted that the values of the new tensile forces generated are small and stable to avoid muscle strain and secondary damage. 

## 4. Experiments and Results

This section conducts numerical simulation experiments for the musculoskeletal arm under the designed neural feedback controller using Euler approximation, as explained in Appendix A. The biophysiological parameters of the musculoskeletal arm model and skeletal muscles are listed in Table 3 and Table 4, separately. There are a total of two experiments carried out, which are the motion test and the robustness test. For the motion test, it aims to verify that the controller can move the arm along a bell-shaped movement trajectory from the start position to the target position. For the robustness test, it aims to verify that the controller can complete the motion tasks regardless of changes in the parameter settings of the arm.

### 4.1. Motion Test

This neural feedback controller is designed for arm reaching movements. Hence, the most important requirement for the controller is that it can control the patient’s arm from the start position to the target position, and it can control the skeletal muscles to generate smooth tensile forces without exceeding the maximum allowable tensile force. The start, target position, and maximum allowable tensile force for each muscle are listed in Table 5. In addition, in order to show the good performance of this controller, an opposite controller is tested, and the results of them are compared. 

**Figure 7:**
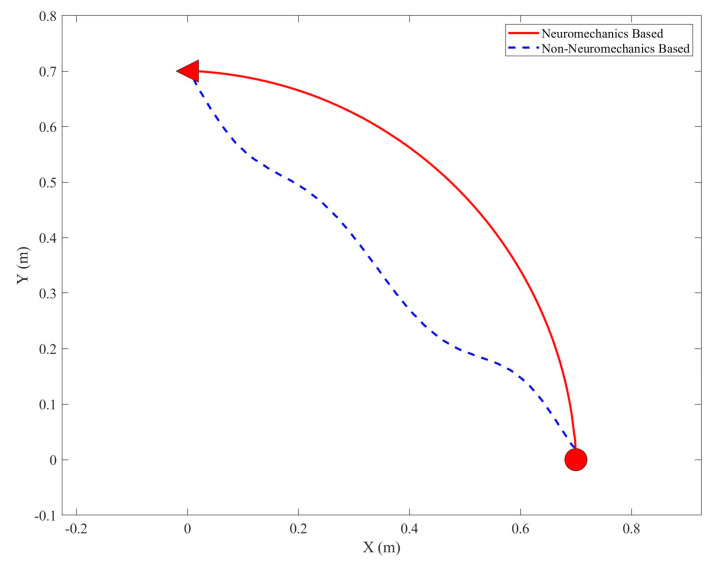
Movement trajectories (start from (0.7,0) and end at (0,0.7)) generated by the musculoskeletal arm under neuromechanics—based and non—neuromechanics—based neural feedback controllers.

**Figure 8:**
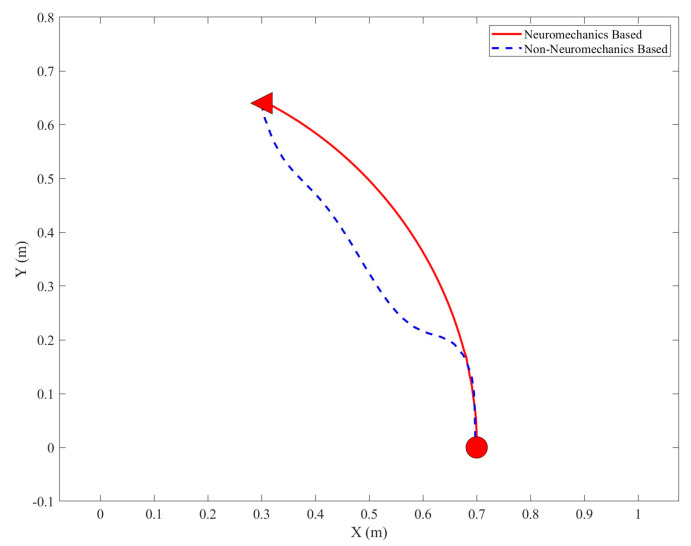
Movement trajectories (start from (0.7,0) and end at (0.3,0.65)) generated by the musculoskeletal arm under neuromechanics—based and non—neuromechanics—based neural feedback controllers.

**Figure 9:**
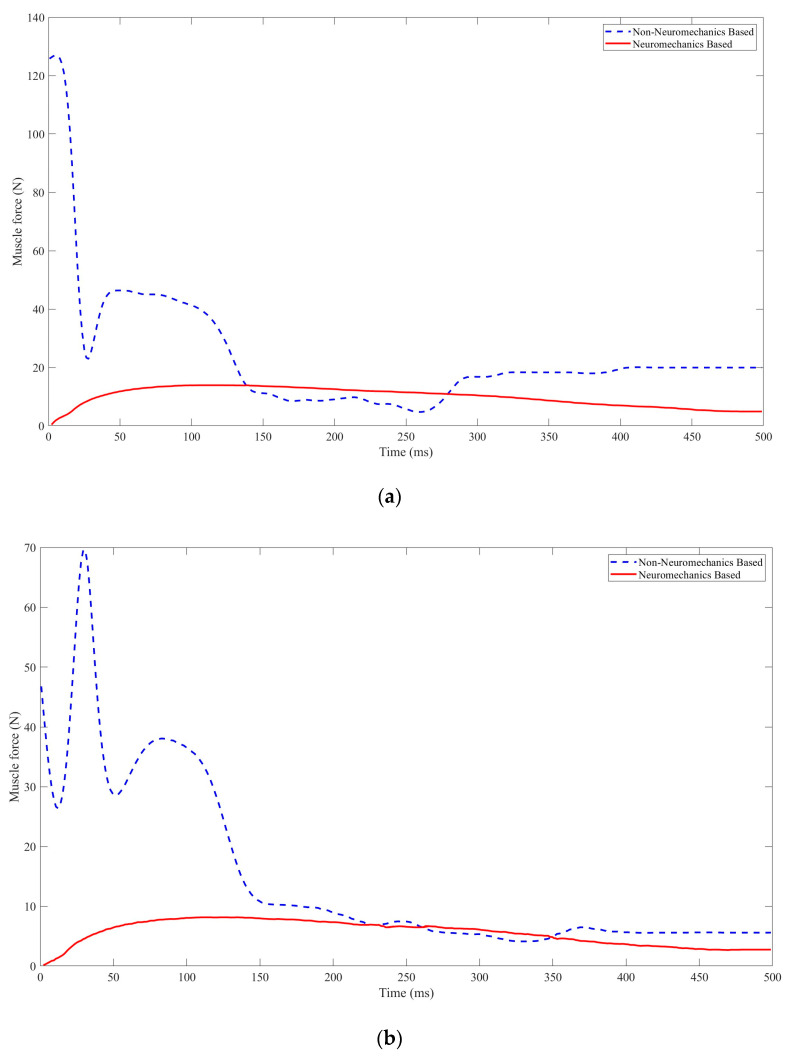

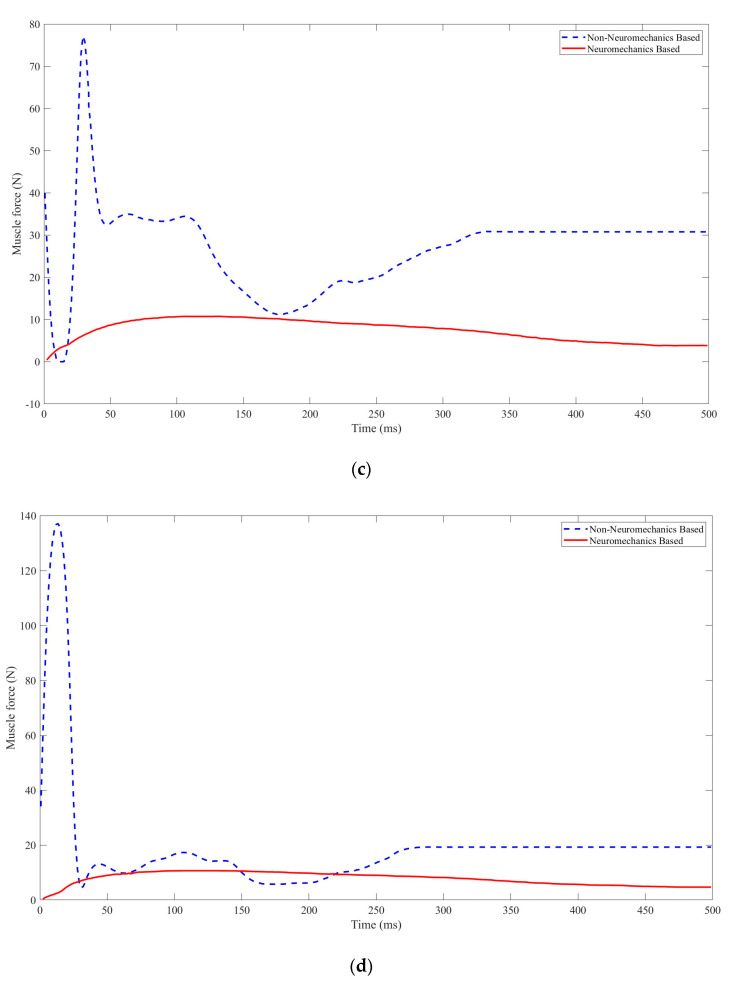

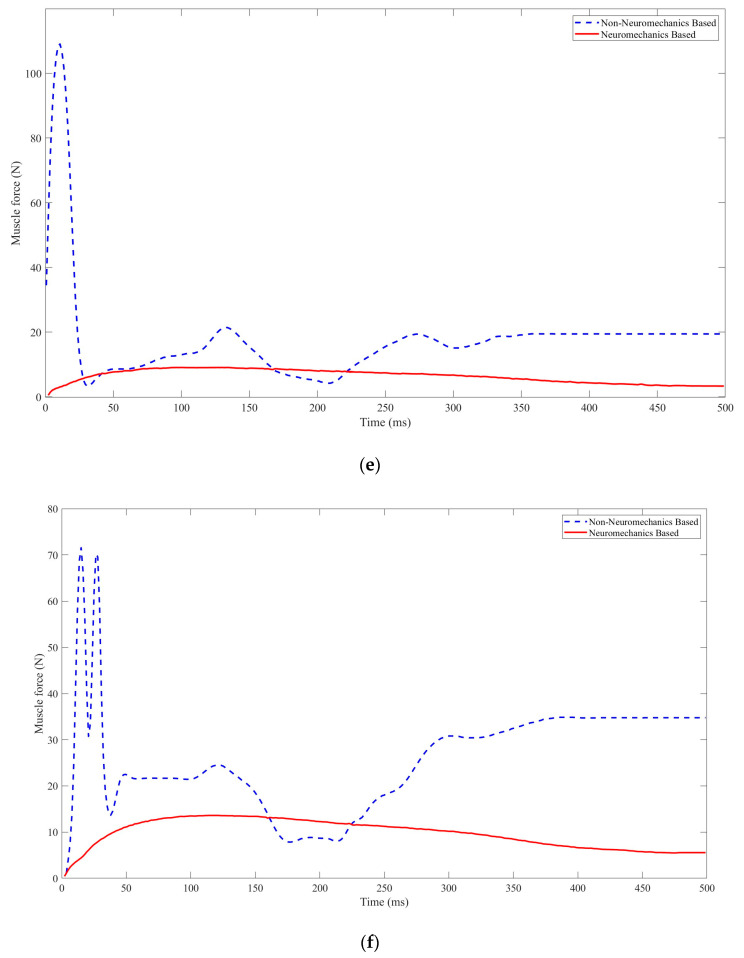

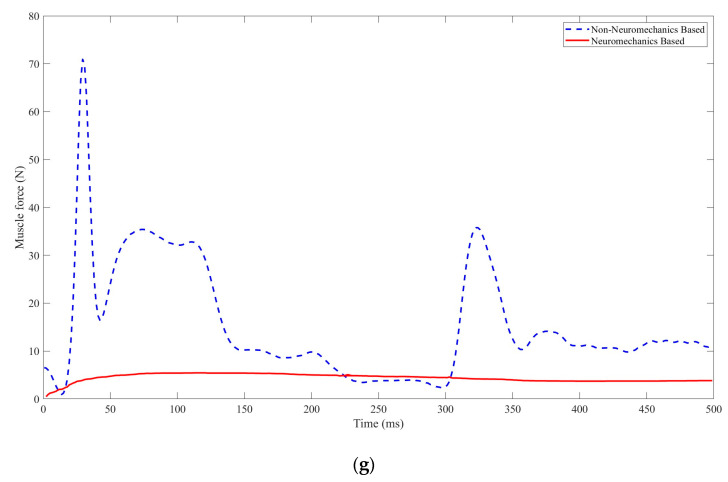
The muscle activity and forces during the arm reaching movements are compared between the neuromechanics—based and non—neuromechanics-based controllers. Subfigures (**a**–**g**) depict the muscular activities of skeletal muscles 1–7 during planar arm reaching movements.

Furthermore, to ensure that the designed neural feedback controller can move the arm from one position to another target position, a new target position is set as x=0.30 m, y=0.65 m, and other parameters are left unchanged. Figure 7 and Figure 8 depict the trajectories of the musculoskeletal arm under the influence of two distinct neural feedback controllers. The results of the comparison between the most important parameter indicators for the reaching movements under these two controllers have been presented in Table 6 and Table 7, respectively. According to the results of the simulation, the neuromechanics-based neural feedback controller can move the human arm to the target position with a bell-shaped trajectory that closely resembles the actual movement trajectory of the human arm, whereas the non-neuromechanics-based neural feedback controller is unable to do so. Based on the findings presented in Figure 9, it is evident that the neuromechanics-based neural feedback controller successfully maintains the tensile forces of each skeletal muscle below the maximum permissible threshold. Notably, the values of these forces exhibit greater stability when regulated by the neuromechanics-based neural feedback controller compared to the non-neuromechanics-based neural feedback controller.

Upon comparing the muscular activities during the reaching movements under the neuromechanics-based or non-neuromechanics-based neural feedback controller, it is evident that the neural feedback controller incorporating neuromechanics results in a smooth muscle force alteration, characterized by a minimal maximum deviation and low peak muscle force. Conversely, the neural feedback controller devoid of neuromechanics leads to a sharp muscle force change, exhibiting a sizable maximum deviation and high peak muscle force, some of which exceeds the maximum peak muscle force of 120 N allowed. In addition, the neuromechanics-based neural feedback controller exhibits a decay degree of up to 1, thereby eliminating oscillations of muscle tensile force during the arm reaching movements. Table 8 provides a summary of the selected performance metrics used to evaluate the arm reaching movements under the controllers, along with their corresponding values.

### 4.2. Robustness Test

**Figure 10:**
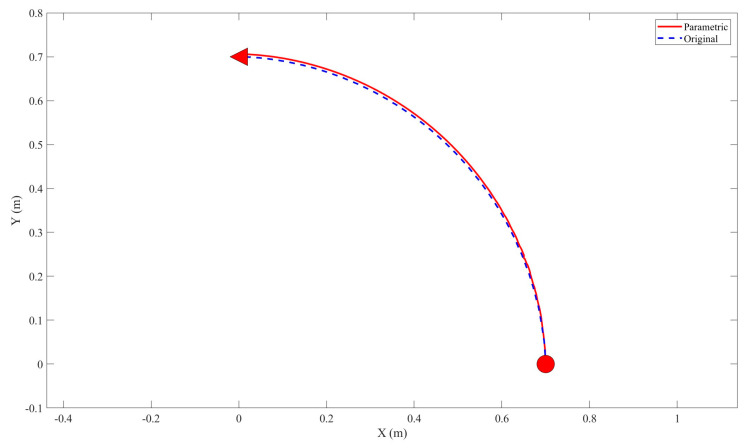
The neuromechanics-based neural feedback controller generates movement trajectories of the musculoskeletal arm with different parameters.

As each patient has unique physiological properties, it is crucial for the neural feedback controller to perform well even when the parameters of the musculoskeletal arm are altered. Hence, this section examines the robustness of the controller to confirm its applicability to different patients. For the robustness test, a 15% uncertainty is added to all the parameters of the musculoskeletal arm, while the motion task remains unchanged. The start position, target position, and maximum tensile force limit for each muscle remain the same. The movement trajectories under the neural feedback controllers with different parameters are depicted in Figure 10, and the comparisons between these two trajectories are listed in Table 9. The difference between the two trajectories is measured by the maximum difference on the x and y axis, which is found to be less than 10% from the experimental results, demonstrating acceptable performance. 

## 5. Conclusions and Discussion

In this paper, a neuromechanics-based neural feedback controller for arm reaching movements is developed. Through conducting the simulation experiments, it is proven to be effective at driving the patient’s arm toward the target position and robust enough to complete different reaching movements tasks. It can also be observed that the controller does not stimulate the skeletal muscle to generate too much tensile force. It means that the patients are protected from experiencing serious muscle strain. To develop a controller with such good performance, first, a musculoskeletal arm model is constructed to simulate the real biomechanical structure of the human arm. Second, based on this model, a neural feedback controller is developed which follows the principles of neuromechanics. In detail, the controller imitates the behavior of the human brain, which consists of three main components: fuzzy system, feedback gains, and neural networks. A fuzzy system is used here to mimic the human brain that cannot determine the precise distance between the current position and the target position but can only approximately compute it. Feedback gains are utilized here since it has already been validated that they change the joint angles and joint angle velocity to fulfil motion tasks. Neural networks are adopted here to learn the actual muscle activity that occurs during the arm reaching movements to ensure that the controller can stimulate the muscles to generate small, steady forces that are sufficient to move the arm from one position to the other. In conclusion, with this neuromechanics-based neural feedback controller, the patient’s arm can be safely driven from the target position to the target position without consuming energy too much due to small and steady muscle forces generated. 

A feasible alternative to the approach adopted in this paper, which models the kinematics and dynamics of the skeletal muscle model, analyzes its system characteristics, and then designs a controller, is to use software already available for skeletal muscle systems to train an efficient controller directly through thousands of unrestricted interactions with the musculoskeletal system. A good example of adopting this method is when Wannawas et al. [36] used neuromechanics-based deep reinforcement learning to control FES during cycling. They first developed a neuromechanical model of cycling based on an open software named “OpenSim”, and then optimized the control strategy via allowing interactions between the musculoskeletal model and the mechanical properties of a bicycle. The results they presented show that their controller can achieve approximately optimal cycling performance. This approach is effective in seeking the near-optimal neural feedback controller. However, because the interactions are based on a very particular model, the performance of the controller is strongly individualized. The trajectories produced by the controller for the upper limb models with different parameter values did not significantly change in the simulation experiments for robustness testing in this paper because only the important parameters of the human upper limb arm are taken into account, providing a tolerance space for system variations.

Although the controller is proven to be effective via numerical simulation experiments, it still has a way to go before it can be used in real life. There are several issues with this gap. First, unlike physical models, which can qualitatively and quantitatively explain a physical occurrence, biological models are unable to do so. The experimental theory of biological simulation cannot be perfectly applied to a real biological scenario since biological models can only provide qualitative explanations for biological phenomena rather than comprehensive quantitative ones. Second, it is challenging to simulate whether the variable amount of delay will have an impact on the control effect because since the nerve signal travels from the brain, stimulates the corresponding muscle, and then produces the corresponding movement, there is frequently a significant time delay between these three events. Third, given that each person’s biological characteristics are unique, even though this paper demonstrates through robustness testing that parameter changes within a specific range will not affect the effectiveness of the controller, when the function of the individual completely changes, the developer must analyze the characteristics of the patient to develop a suitable controller. Therefore, this investigation requires a lot of human and material investment, which is obviously detrimental to the business. Despite the difficulties mentioned above, it cannot be denied that the goal of this paper is to propose a novel, highly effective controller based on neuromechanics, developed specifically for human upper limb planar movements, and to demonstrate a potential solution to the problem of patients who are unable to actively control their movements.

## Figures and Tables

**Table 1:** Fuzzy rule base table for proportional gain $\Delta K_{p}$.

$\Delta K_{p}\;\left(e,\Delta e\right)$		e						
		NB	NM	NS	ZO	PS	PM	PB
Δe	NB	ZO	PS	PS	PM	PM	PB	PB
	NM	ZO	ZO	PS	PM	PM	PB	PB
	NS	NM	NS	ZO	PS	PM	PM	PM
	ZO	NM	NM	NS	ZO	PS	PS	PM
	PS	NM	NM	NS	NS	ZO	PS	PS
	PM	NB	NM	NM	NM	NS	ZO	ZO
	PB	NB	NB	NM	NM	NS	NS	ZO

**Table 2:** Fuzzy rule base table for proportional gain $\Delta K_{d}$.

$\Delta K_{p}\;\left(e,\Delta e\right)$		e						
		NB	NM	NS	ZO	PS	PM	PB
Δe	NB	ZO	PS	PS	PM	PM	PB	PB
	NM	ZO	ZO	PS	PM	PM	PB	PB
	NS	NM	NS	ZO	PS	PM	PM	PM
	ZO	NM	NM	NS	ZO	PS	PS	PM
	PS	NM	NM	NS	NS	ZO	PS	PS
	PM	NB	NM	NM	NM	NS	ZO	ZO
	PB	NB	NB	NM	NM	NS	NS	ZO

**Table 3:** Biophysiological parameters of the musculoskeletal arm model.

Parameter	Upper Limb	Forearm
$\mathrm{Length}\;L_{i}\;\left[m\right]$	0.2980	0.4190
$\mathrm{Mass}\;m_{i}\;\left[\mathrm{kg}\right]$	2.0890	1.9120
$\mathrm{Mass}\;\mathrm{Centre}\;\mathrm{Length}\;L_{gi}\;\left[m\right]$	0.1520	0.1810
$\mathrm{Inertia}\;\mathrm{Moment}\;I_{i}\;\left[\mathrm{kg}\;\cdot m^{2}\right]$	0.0159	0.0257
Joint Viscosity Matrix V [Nms/rad]	$\left[\begin{array}{cc} 0.74 & 0.1\\ 0.1 & 0.82 \end{array}\right]$	

**Table 4:** Biophysiological parameters of skeletal muscles.

Muscle	Insertion Position [m]	
1	$a_{1}=0.055$	$b_{1}=0.080$
2	$a_{2}=0.055$	$b_{2}=0.080$
3	$a_{3}=0.030$	$b_{3}=0.120$
4	$a_{4}=0.030$	$b_{4}=0.120$
5	$c_{51}=0.040$	$c_{52}=0.045$
6	$c_{61}=0.040$	$c_{62}=0.045$
7	$c_{71}=0.040$	$c_{72}=0.030$

**Table 5:** Parameter settings of simulation experiments.

Parameter	Value
Start Position (x,y)	(0.70, 0.00) [m]
Target Position (x,y)	(0.00, 0.70) [m]
Maximum Muscle Tensile Force	120 [N]

**Table 6:** Comparison I of the effectiveness of neural feedback controllers that are based on neuromechanics and those that are not.

	Minimum Absolute Deviation [m]	Average TimeConsumption [ms]
Neuromechanics Based	0.000651	426
Non-Neuromechanics Based	0.086500	761

**Table 7:** Comparison II of the effectiveness of neural feedback controllers that are based on neuromechanics and those that are not.

	Minimum Absolute Deviation [m]	Average TimeConsumption [ms]
Neuromechanics Based	0.000768	459
Non-Neuromechanics Based	0.059500	821

**Table 8:** Selected metrics for evaluating the performance of arm reaching movements under non—neuromechanics—based (NNB) and neuromechanics—based (NB) neural feedback controllers.

	Muscle 1		Muscle 2		Muscle 3		Muscle 4		Muscle 5		Muscle 6		Muscle 7	
	NNB	NB	NNB	NB	NNB	NB	NNB	NB	NNB	NB	NNB	NB	NNB	NB
Rise time [ms]	130.0	16.3	29.3	3.4	23.6	16.5	26.4	19.5	24.7	13.5	9.2	19.1	19.9	28.2
Settling time [ms]	404.0	483.0	275.5	455.9	348.6	460.2	297.1	477.0	355.7	471.7	382.9	461.7	491.7	361.4
Peak value [N]	127.1	13.8	69.5	8.1	77.1	10.7	136.7	10.6	109.0	9.1	71.5	13.6	70.9	5.4
Overshoot [N]	107.0	8.9	63.9	5.4	46.4	6.9	117.5	5.9	89.5	5.7	36.8	8.1	59.9	1.6
Number of oscillations	2	0	3	0	3	0	3	0	3	0	3	0	3	0
Oscillation period [ms]	48.7	0	54.5	0	35.8	0	31.6	0	121.7	0	11.4	0	42.9	0
Degrees of attenuation	0.646	1	0.450	1	0.549	1	0.904	1	0.804	1	0.020	1	0.502	1

**Table 9:** Comparison between the trajectories performed by the musculoskeletal arm model with different parameters.

	Minimum Absolute Deviation [m]	Average Time Consumption [ms]
Neuromechanics Based	0.000768	459
Non-Neuromechanics Based	0.059500	843

## Data Availability

The data that support the findings of this study are available on request from the corresponding author.

## Appendix A

Euler approximation of a differential equation of the planar musculoskeletal arm is as follows:

(A1) $$M\left(\theta \right)\ddot{\theta }+C\left(\theta ,\dot{\theta }\right)\dot{\theta }+V\dot{\theta }=\tau$$

Following the Euler method, the above equation can be rewritten as follows:

(A2) $$\{\begin{array}{c} \dot{\theta }=\frac{d\theta }{dt}\\ \frac{d^{2}\dot{\theta }}{dt^{2}}=M^{-1}\left(\theta \right)\left[\tau -C\left(\theta ,\dot{\theta }\right)\dot{\theta }-V\dot{\theta }\right] \end{array}$$

thus yielding

(A3) $$\{\begin{array}{c} \dot{\theta }\left(t\right)=\dot{\theta }\left(t-dt\right)+dt\cdot M^{-1}\left(\theta \left(t-dt\right)\right)\left[\tau -C\left(\theta \left(t-dt\right),\dot{\theta }\left(t-dt\right)\right)\dot{\theta }\left(t-dt\right)-V\dot{\theta }\left(t-dt\right)\right]\\ \theta \left(t\right)=\theta \left(t-dt\right)+dt\cdot \dot{\theta }\left(t-dt\right) \end{array}$$
